# Poisoning by Arsenic and Laudanum Combined, in Which the Symptoms Usually Produced by Arsenic Were Absent

**Published:** 1830-10

**Authors:** Egerton A. Jennings

**Affiliations:** Member of the Royal College of Surgeons, &c.


### POISONING BY ARSENIC AND LAUDANUM.

*Poisoning by Arsenic and Laudanum combined, in which the Symptoms usually produced by Arsenic were absent.* By EGERTON A. JENNINGS, F.L.S. Member of the Royal College of Surgeons, &c.

ON Saturday, August the 7th, I was requested to visit Marianne Warwick, who was reported to have taken poison. It appeared from the girl's own statement, that she had taken about three ounces of laudanum and a quantity of arsenic, which subsequent inquiry proved to be about two drachms. She took the poison about twelve o'clock, and I saw her soon after four. She had no pain in the stomach, bowels, or head; had no heat or burning in the throat; complained of no uneasiness when the abdomen was pressed upon, and was perfectly collected. She complained, however, of feeling tired and sleepy, which she said was occasioned by her having taken an emetic two hours before I saw her, which had acted violently and tired her very much. The only symptoms which she had, that were at all indicative of her having taken poison, were that her eyes were bloodshot and heavy, and the pupils contracted; she was rather disposed to sleep, the pulse was about 100, and the vomiting continued, perhaps, a little longer than might be expected from the effect of the emetic. The symptoms at no period prior to my seeing her had been more marked than at that time. In fact, so completely were all symptoms of poisoning by arsenic absent, that a physician and surgeon, who saw her in about an hour after she had taken the poison, and attended to her until I saw her, could not persuade themselves that she had taken poison at all, though the girl had stated such to be the fact.

My first effort was to get the stomach freely evacuated, taking care to preserve what was rejected for future analysis, as what had been previously vomited had unfortunately been thrown away. The stomach being well emptied, my attention was directed to counteract the effects of the laudanum, as the only marked symptoms were the effects of its action upon the brain. The usual medicines, together with bleeding from the jugular vein, leeches, blisters, and cold affusion, were employed. The patient was kept constantly walking about. No alteration in the symptoms took place before half-past seven o'clock; excepting that she complained of being more drowsy, and with greater difficulty was kept awake, frequently dropping asleep, even while walked about. About half-past seven o'clock she once or twice complained of some pain in the bowels, but there was no tenderness on pressure, and no pain in the stomach. The bowels acted once, very comfortably, about this time. At eight o'clock she sunk into a state of coma, with dilated pupils and laborious breathing; before nine she died.

*Examination of the body, sixteen hours after death.* External appearance of the body healthy, abdomen not swelled. On removing the calvarium, more blood than natural was discharged from the vessels connecting it with the dura mater. The sinuses were loaded with dark blood: the dura mater was very vascular. On removing the dura mater, the parts presented a very turgid appearance, and the large veins which wind between the convolutions of the brain, instead of presenting their ordinary blue tint, were of a dirty treacle colour. The pia mater and arachnoid were healthy, but the former was more than usually loaded with blood. On cutting into the substance of the brain, the number of bloody points, from the division of vessels, was much greater than usual, and they presented the same dirty colour which I have endeavoured to describe above. The ventricles contained no fluid. The substance of the brain throughout was remarkably firm and healthy, and there was no effusion or other disease discovered in any part of it.

On opening the cavity of the abdomen, the stomach appeared large, and on its surface a few small vessels were evident in one or two places, but nothing that would have attracted attention in an ordinary case. The lower part of the ileum presented several patches, where the vessels were finely injected and of a bright vermilion colour. The other organs in the abdomen presented, externally, no unhealthy appearance.

On opening the stomach, it was found to contain about half a pint of fluid, which was saved for analysis. Its mucous coat was generally rather pale, but at the great arch, near the pylorus, there were two patches which presented a red appearance; one about the size of a half-crown, the other about as large as a shilling. There was no ulceration, and, when the stomach was held to the light, no part of it appeared at all thinner than natural. In fact, the stomach presented no unnatural appearance, excepting the two red patches I have already mentioned, and very strongly marked rugæ, which were fully as prominent as those represented by Dr. Baillie, in fig. 2, plate 5, of the third fasciculus of his engravings of Morbid Anatomy.

The small intestines being removed from the body, and their contents collected, they were slit up in their whole course, so as to expose their inner coat. In the duodenum, the mucous membrane presented throughout a general light pink appearance. In the jejunum it was highly injected, and presented numerous patches of an intensely red colour. In the ileum the appearances of inflammation were less marked than in the jejunum, but it presented several patches acutely inflamed. These patches shone through the thin coats of the intestine, and occasioned the vascular appearance that had been observed through the peritoneal coat. No ulceration of the small intestines could be detected.

The cœcum and colon were healthy. There were not any ulcers in the rectum, nor any other disease of that part.

In the chest there was no appearance of disease, unless we describe as such a rather flaccid state of the heart, and a light leaden hue on the surface of the lungs. The large vessels about the heart were not more loaded than they are generally found, but the blood in them also was very dark, and appeared much loaded with carbon.

On analysis, the contents of the stomach exhibited a slight trace of arsenic. Sulphuretted hydrogen gas gave the fluid a yellow tinge. Dr. Marcet's test with the nitrate of silver and ammonia threw down a very slight yellow precipitate, but no quantity of precipitates could be collected; from which I assume that the stomach had been almost completely emptied of the arsenic.

From the fluid contained in the small intestines, a sufficient quantity of precipitates could be collected to reduce the arsenic to its metallic state.

The fluid vomited at half-past four o'clock contained so much arsenic, that, from one half of it, many grains of the precipitates were collected, a large quantity of metallic arsenic reduced, and a very well-marked alloy formed with it and copper.

From the fact of arsenic being in the bowels, it is certain that that poison had been taken. From the girl's own statement, there can be no doubt of her having taken it about twelve o'clock; and, from the large quantities found in the fluid vomited, it is evident that a large quantity remained in the stomach for four hours: and yet, during the whole of this time, she presented no symptoms of poisoning by arsenic. In fact, if we refer to ORFILA's work on Poisons, we shall find that there was not one symptom of poisoning by arsenic which he enumerates, if we except vomiting; and the vomiting did not come on until two hours after the arsenic had been taken, and after an emetic with sulphate of zinc had been given; and the fluid vomited was neither brown nor bloody, as he describes, but appeared to be a mixture of the fluid swallowed and mucus. Even to the hour of death, excepting pain in the bowels, complained of once or twice, above seven hours after the poison had been taken, there was no symptom of poisoning by arsenic.

The appearances of the stomach on dissection were as unsatisfactory as the symptoms. The great arch, near the pylorus, from being the most depending part of the organ, is very frequently found on dissection to be red, from the blood settling in that part after death; and the vascularity in this case was not at all such as would have attracted attention under ordinary circumstances. As we trace the course of poison in the intestines, the appearances become more marked. In the jejunum the appearances of inflammation were very strongly marked, and in the ileum also; yet in the duodenum, through which the whole of the poison must have passed that entered the jejunum, they were much less apparent.

In the above case there can be no doubt that the patient died from the effects of the laudanum: it being certain that the girl took three ounces of laudanum, and the symptoms corresponding with the effects of that drug: but still, in connexion with medical jurisprudence, several interesting inquiries present themselves.

1st. After so large a quantity of laudanum had been taken, why did not more urgent symptoms of that poison present themselves at an earlier period? I think it must be acknowledged that four hours was a greater length of time than would be expected to elapse, after taking three ounces of laudanum, before decided symptoms presented themselves. During this time, too, it appears that sufficient opium was absorbed to occasion death; for neither in the fluid vomited after four o'clock, nor in the contents of the bowels, could any smell of laudanum be detected. Did the arsenic, by acting as a stimulant, counteract for a time the sedative effects of the opium? or did the arsenic, by occasioning constant effusion of fluid from the mucous coat of the stomach, render its absorption less rapid?

2d. Why did the arsenic appear to produce no effect until it had passed into the intestines, and even then no marked effect until it had proceeded as far as the jejunum? Did the opium render the stomach so far insensible to the action of the arsenic as to prevent it from producing inflammation of the organ? For inflammation we should naturally expect to find, after arsenic had remained in the stomach in considerable quantities for above four hours, which it must have done in this case, from the quantity vomited after four o'clock. The opium, I apprehend, must have produced this effect: for, when the arsenic had proceeded as far as the jejunum, which probably had not been acted upon by the opium, we find the parts acutely inflamed, and at half-past seven in the evening the patient complained of pain in the bowels.

That arsenic does not always produce pain, I am aware. Several cases are on record in which pain did not occur: but I do not remember to have met with any case in which arsenic and laudanum in large doses had both been taken, where the symptoms of arsenic have all of them been so completely wanting, and where the appearances on dissection have been exactly similar. I have therefore thought this case worth placing upon record.

*Leamington Spa; August* 16, 1830.

